# A Case Report of Sepsis Secondary to Perforated Cholecystitis in the Presence of Severe Aortic Stenosis: Diagnosis and Management

**DOI:** 10.7759/cureus.60382

**Published:** 2024-05-15

**Authors:** Austin Rahman, Taylor E Collignon, Jason Smith

**Affiliations:** 1 Emergency Medicine, Lake Erie College of Osteopathic Medicine, Bradenton, USA; 2 Internal Medicine, Lake Erie College of Osteopathic Medicine, Bradenton, USA; 3 General Surgery, AdventHealth Florida, Tavares, USA

**Keywords:** gallblader disease, severe sepsis, aortic stenosis, cholecystitis, perforated gallbladder

## Abstract

Gallbladder perforation is a rare complication of acute cholecystitis that is associated with significant morbidity and mortality. Many cases of gallbladder perforation are not diagnosed until surgery, as the physical symptoms closely mimic acute cholecystitis. Gallbladder perforation is most common among older males with associated comorbidities, and preoperative assessment of comorbidities, particularly cardiac, is critical to determine the appropriate clinical course. We report a case of a 77-year-old male who presented initially with low blood pressure and right upper quadrant pain (RUQ) after not feeling well for five days. CT of the abdomen/pelvis with IV contrast demonstrated acute perforated cholecystitis, and general surgery was consulted for a cholecystectomy. Due to the patient’s past medical history of severe aortic stenosis (AS), cholecystectomy was deferred and a cholecystostomy tube was placed by interventional radiology. This report aims to provide an example of a case of perforated cholecystitis with sepsis and how it can be diagnosed and managed non-surgically in the presence of pre-existing severe AS.

## Introduction

Acute cholecystitis poses significant risk due to several serious complications, including gangrenous cholecystitis, hemorrhagic cholecystitis, emphysematous cholecystitis, gallstone ileus, and gallbladder perforation [1]. Gallbladder perforation is a rare complication that occurs due to a bladder wall defect, and it has an estimated incidence of 2% of all gallbladder pathologies [2]. The most common site of gallbladder perforation is the fundus, as it is the most distal part of the blood supply to the organ [3]. Risk factors for gallbladder perforation include male sex, advanced age, medical comorbidities, cholelithiasis, cholecystitis, and trauma [4]. 

Gallbladder perforation has been classified into three types by Neimeier. Type I is known as bilio-enteric fistulae and represents chronic gallbladder perforation with fistulous formation to surrounding tissue. Type II occurs when there is subacute perforation causing the formation of localized cholecystic abscess(es) and localized peritonitis symptoms. Lastly, type III is when acute perforation into the peritoneal cavity causes generalized peritonitis [5]. 

Aortic stenosis (AS) is a very common cardiac pathology and is more frequently seen in patients who have a history of smoking, hypertension, and hyperlipidemia. It is due to valve leaflet calcification from inflammation and lipid accumulation and causes the valve to be less flexible and become rigid [6]. This adds pressure on the left ventricle, particularly when paired with pre-existing hypertension, and this can cause significant left ventricular hypertrophy. Patients with severe AS have an increased risk of major coronary adverse events during surgery, and this is proposed to be due to tachycardia and hypotension during anesthesia that may lead to decreased coronary perfusion [7]. Therefore, in patients undergoing non-cardiac procedures, preoperative assessment of the severity of AS is critical to determine the appropriate clinical course. 

Here, we present a case of perforated cholecystitis found in a 77-year-old male patient who originally presented with severe sepsis and right upper quadrant (RUQ) pain. We demonstrate that this diagnosis can be managed non-surgically with a percutaneous cholecystostomy tube when the patient has underlying comorbidities that limit surgical intervention, such as severe AS.

## Case presentation

A 77-year-old male with past medical history (PMH) of hypertension, AS, coronary artery disease (CAD), benign prostatic hyperplasia (BPH), and diabetes mellitus type 2 presented to the emergency department (ED) with complaints of low blood pressure after feeling unwell for the previous five days. He also had complaints of RUQ pain. In the ED, his blood pressure was 80/46, and his heart rate was 100. The patient and his wife reported that he was having chest pain that he attributed to indigestion five days prior, and it improved after taking sublingual nitroglycerin. However, he woke up that evening with nausea and vomiting. He had decreased appetite since then and developed a fever and chills a day before his ED visit. His systolic blood pressure was in the 80s, so he contacted his cardiologist, who told him to seek care in the ED. He had no family history on file and no known allergies. Past surgical history included four stents placed previously for his CAD and a drug-eluting stent to the left anterior descending (LAD) secondary to AS. He also had severe AS, with his last echo 10 days prior showing an aortic valve area of 0.9 and intact ejection fraction of 65-69%, and he was undergoing transcatheter aortic valve replacement (TAVR) workup. On the initial physical exam, he was awake and alert, appeared obese with a protuberant abdomen, and had abdominal tenderness in the RUQ without rebounding or guarding (positive Murphy's sign).

The initial workup showed notable results: white blood cells (WBCs) 15.5, 20% bands, hemoglobin (Hgb) 13.7, platelets 158, Na 130, K 3.2, blood urea nitrogen 22, creatinine 1.3, glucose 117, aspartate transaminase (AST) 191, alanine transaminase (ALT) 414, alkaline phosphatase 404, lipase 45, troponin T 46, and D-dimer 6.88 (Table 1). 

**Table 1:** Lab values

Complete blood count	Reference values	Admission values
White blood cells	4.40-10.50 10^3^/uL	15.5
Red blood cells	4.00-5.5.65 10^6^/uL	4.53
Hemoglobin	12.6-16.7 g/dL	13.7
Hematocrit	36.9-48.5%	40.1
Mean corpuscular volume	82.4-99.3 fL	88.5
Mean corpuscular hemoglobin	27.5-34.1 pg	30.2
Mean corpuscular hemoglobin concentration	31.7-36.1 g/dL	34.2
Red cell distribution width	11.4-14.9%	13.2
Platelet count	139-361 10^3^/uL	158
Mean platelet volume	9.7-12.5 fL	10.4
Neutrophil %	50-70%	67.6
Lymphocyte %	1-15%	3.9
Monocyte %	1-15%	2
Comprehensive metabolic panel		
Sodium	135-145 mmol/L	130
Potassium	3.5-5 mmol/L	3.2
Chloride	98-110 mmol/L	93
Carbon dioxide	24-31 mmol/L	19
Anion gap	5-15 mmol/L	18
Blood urea nitrogen	5-25 mg/dL	22
Creatinine	0.6-1.20	1.30
Glucose	70-100 mg/dL	117
Calcium	8.5-10.5	8.4
Aspartate transaminase	4-46 U/L	191
Alanine transaminase	4-51 U/L	414
Alkaline phosphatase	40-129 U/L	404
Protein, total	6.5-8.0 g/dL	6.7
Albumin	3.20-5.50 g/dL	3.20
Bilirubin, total	0.10-1.5 mg/dL	1.80
Biomarkers		
Aminoterminal pro B-type natriuretic peptide	0-1,800 pg/mL	2,612
Lactate	0.5-2.20 mmol/L	2.0
D-dimer	<0.50 mg	6.88

**Figure 1:**
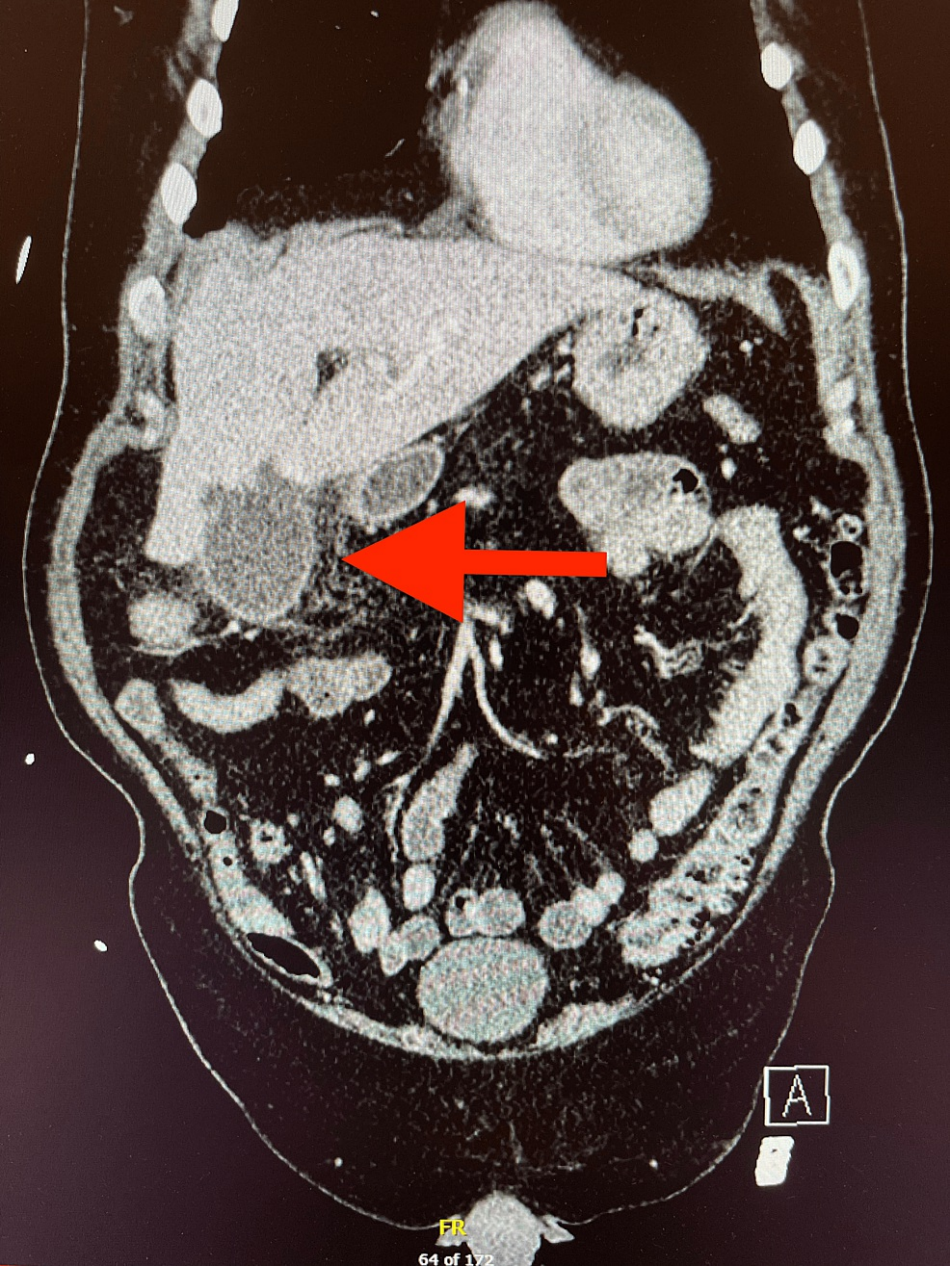
CT scan of the abdomen with and without contrast The arrow points to show perforated gallbladder.

Urinalysis was not noteworthy, and no source of infection was indicated. EKG findings were also benign. A computed tomography angiogram (CTA) of the chest was negative for pulmonary embolism with mild bibasilar atelectasis and right-sided pleural effusion with no infiltrate. Chest X-ray showed no acute intrathoracic disease. Computed tomography (CT) of the abdomen/pelvis with IV contrast demonstrated acute perforated cholecystitis with several small hepatic micro-abscesses (Figure 1) and mild mucosal edema of the proximal and mid duodenum and a short segment of the hepatic flexure likely reactive in etiology.

A diagnosis of severe sepsis secondary to perforated cholecystitis was made, and the patient was escalated to the intensive care unit (ICU). His home hypertensive medications were held, and his blood pressure was monitored closely without vasopressors. He was given Rocephin and Zosyn x1 and 2,571 ml of lactated ringer solution. General surgery was consulted for cholecystectomy the following morning.

**Figure 2:**
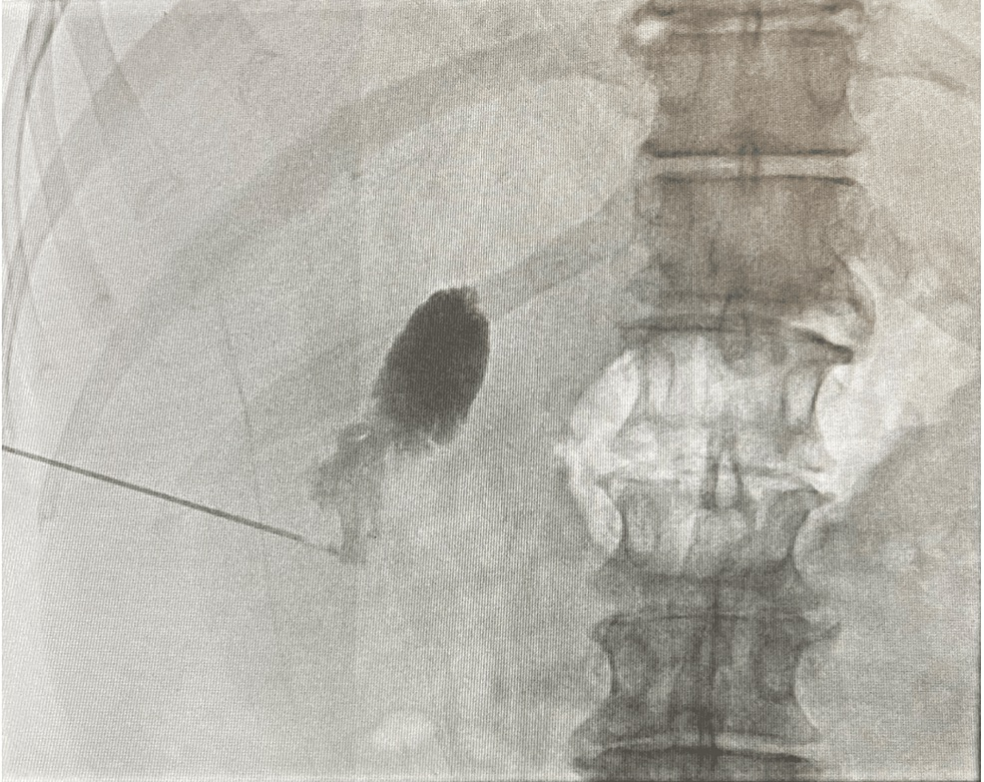
CT-guided cholecystostomy tube (IR)

On hospital day 2, he had normal sinus rhythm to sinus bradycardia, was put on nothing by mouth status (NPO), and was receiving Levophed 0.5, lactated ringer’s 75, and 3-5 L oxygen on nasal cannula. He reported no abdominal pain or nausea and was alert and oriented x3. After a review of the patient's chart, due to the patient’s history of severe AS, it was discussed by general surgery and interventional radiology to plan a percutaneous cholecystostomy drain to stabilize the patient and then transfer care to a tertiary hospital. The CT-guided cholecystostomy tube (Figure 2) was placed on hospital day 2, and it was discussed with the patient that surgical intervention would need to wait at least six weeks to allow reduction of inflammation and to stabilize the gallbladder for removal. A body fluid culture revealed heavy growth of *Escherichia coli*, and he was continued on Zosyn and was given a dose of tobramycin.

The patient did well postoperatively. He had one episode of emesis the night of the procedure, and his nausea was controlled with ondansetron. On admission day 3, he reported feeling uncomfortable abdominal distension but denied pain. He was hemodynamically stable without vasopressors, and his blood pressure was trending hypertensive. His renal function improved, and he had downward-trending liver function tests. While in the ICU, he developed vomiting and abdominal distension. On abdominal and pelvis X-rays (KUB), gastric distension was noted and was suspected to be developing ileus, so a nasogastric (NG) tube was placed.

On hospital day 4, he felt much better and reported no abdominal pain or nausea. His abdomen was less distended, and he tolerated clear liquids but had no documented bowel movement. His leukocytosis had resolved, he was afebrile, and he continued his seven-day Zosyn plan. His hypertensive and CAD medications (metoprolol, aspirin/plavix) were restarted, but his statin was held due to transaminitis. An outpatient transcatheter aortic valve replacement (TAVR) follow-up was scheduled.

On admission day 5, the patient was much improved and resumed all home medications. His Zosyn was changed to Unasyn through a midline for 10 days. The surgeon cleared him for discharge and instructed him to follow up with general surgery on discharge. The patient was advised to meet with TAVR, have his AS managed and treated, and then undergo possible gallbladder resection.

## Discussion

This case highlights the rarity of gallbladder perforation with the added misfortune of severe AS. Gallbladder perforation is a rare phenomenon that does not happen commonly [8]. The perforation is often a complication of acute cholecystitis [8,9]. The underlying mechanism behind why the perforation happens is poorly understood because of the rarity of the disease; however, it can be inferred that it is likely due to pressure build-up [9]. Our patient has an extensive medical history of CAD, so it can also be inferred that his vasculature may have been weakened, which may have predisposed him to the perforation. It should also be noted that the perforation does not have to be caused by gallbladder stones, an often underlying cause of acute cholecystitis [9]. Because the complication is a rare effect of cholecystitis, it should not be a lead diagnosis; however, it should be in the back of the physician's mind when making clinical judgment.

Our patient presented with Murphy signs and severe systemic inflammatory response (SIRS) criteria and was hemodynamically unstable. Upon these findings, the lead diagnosis at the time was severe acute cholecystitis. The patient’s worrisome clinical picture expedited the need for a CT abdomen with contrast, although much of the medical literature teaches that acute cholecystitis should be screened first with an ultrasound of the RUQ.

It should be noted that a limitation of this case study was that no ultrasound was done to visualize the gallbladder and if stones were present. The CT scan did not show any stones. Many cases of gallbladder perforation go unnoticed until surgery, as the physical symptoms closely mimic acute cholecystitis unless sepsis ensues. Abdominal pain and distension, high fever, nausea, and vomiting are general symptoms that gallbladder perforation may present with; nevertheless, these symptoms are very common among a wide range of differential diagnoses [8]. Since physical and laboratory exam findings may not provide diagnostic criteria, imaging techniques are helpful in initial diagnosis. Recent findings suggest ultrasound may not be the most reliable choice in diagnosing gallbladder perforation. It is reported that the sonographic hole sign was the only reliable sign of perforation on ultrasound [10]. Standard pelvic CT is more accurate and shows gallbladder wall thickness, defects, free intraperitoneal fluid, pericholecystic fluid, and abscesses [8]. While CT is a more accurate diagnostic tool compared to ultrasound for gallbladder perforation, it cannot rule out perforation until surgery is performed [11]. 

The complexity of this case was furthered by severe AS measuring 0.9 cm. The current literature states that patients with AS less than 1 cm [12] or symptomatic (with variables) should be treated [12]. The underlying causes of AS are vast, but it is a marker of increased morbidity and can be a severe complication to patients undergoing surgical procedures. As such, its importance in management is critical in the care of the patient. The standard of care for this pathology is an aortic valve replacement [12], which was scheduled to be done before the events of the gallbladder perforation. AS is a contraindication to non-emergency surgeries as it predisposes a patient to a multitude of risks where mortality is high [7].

Overall, due to the underlying AS, the patient was not a candidate for urgent gallbladder resection. Thus, the medical team agreed that a stabilization protocol should be made until the patient was deemed to be surgically fit for gallbladder removal. Gallbladder perforations are graded on a scale known as Neimeier, numerically based from 1 to 4 [5,13]; the patient in this case study was classified as 2. The current literature states that perforated gallbladders can become a medical emergency, and thus, cholecystectomy is the first line measure [13] if the perforation meets certain criteria. However, prior case studies have shown that percutaneous gallbladder drainage is an available measure that can temporize until a cholecystectomy can be done [14]. The medical team agreed that an interval of six weeks is necessary for the drainage to reduce inflammation and provide a stabilizing measure to the patient. Interestingly, a case series showed that percutaneous drainage might not only be a stabilizing treatment but also remove the need for surgery in certain patient populations, especially those deemed to be high risk [14].

The rarity of gallbladder perforation confounded with severe AS plays a critical role in the management of the patient. Surgery is likely to be planned for this patient, but the pivotal question that remains is whether a TAVR procedure should be done before gallbladder resection if needed. Research has shown that a delay in TAVR procedures correlates with advanced mortality [15]. Given that perforated gallbladder can also lead to a host of problems and a recurrence of sepsis, the road of treatment for this patient may be variable. The patient’s TAVR team was also consulted. The patient was requested to be seen back in the office after six weeks to see if the gallbladder spontaneously closed or the next best management step. It has been shown in prior studies with cholecystostomy tubes that spontaneous closure has happened [14]. A hepatobiliary iminodiacetic acid (HIDA) scan and ultrasound are among the management options upon follow-up. The need for a multidisciplinary approach quickly became a theme in managing and stabilizing this patient.

## Conclusions

This interesting case highlights the importance of multidisciplinary approaches to complex and rare cases. The authors of this report believe that more case studies regarding gallbladder perforation should continue to be published so that more information regarding management, and treatment options can be readily available to further medical knowledge. Oftentimes, comorbidities may challenge traditional approaches to medicine, and as such, it is imperative for novel cases to be published. Clinicians should continue to be vigilant of classical symptoms of gallbladder pathologies, such as acute cholecystitis or ascending cholangitis, as these are common emergent diseases associated with the gallbladder. Complications regarding these diseases such as a perforation should be on the physician's list of differentials. Proper management of rare diseases is paramount in the quality of healthcare delivered to the patient, and as such, regardless of a hospital being rural or not, physicians should continue to be advocates of their clinical skills by learning and knowing how to deal with rare diseases.
